# Logistic regression analysis of ultrasound findings in predicting the malignant and benign phyllodes tumor of breast

**DOI:** 10.1371/journal.pone.0265952

**Published:** 2022-03-24

**Authors:** Tingting Li, Yanjie Li, Yingqi Yang, Juan Li, ZiYue Hu, Lu Wang, Wei Pu, Ting Wei, Man Lu

**Affiliations:** Department of Ultrasound, Sichuan Cancer Hospital Institute, Sichuan Cancer Center, School of Medicine, University of Electronic Science and Technology of China, Chengdu, China; Tanta University Faculty of Medicine, EGYPT

## Abstract

**Objective:**

To evaluate ultrasound characteristics in the prediction of malignant and benign phyllodes tumor of the breast (PTB) by using *Logistic* regression analysis.

**Methods:**

79 lesions diagnosed as PTB by pathology were analyzed retrospectively. The ultrasound features of PTB were recorded and compared between benign and malignant tumors by using single factor and multiple stepwise *Logistic* regression analysis. Moreover, the *Logistic* regression model for malignancy prediction was also established.

**Results:**

There were 79 patients with PTB, including 39 benign PTBs and 40 malignant PTBs (33 borderline PTBs and 7 malignant PTBs by pathologic classification). The area under the ROC curve (AUC) of lesion size and age were 0.737 and 0.850 respectively. There were significant differences in age, lesion size, shape, internal echo, liquefaction, and blood flow between malignant and benign PTBs by using single-factor analysis (*P*<0.05). Age, internal echo, and liquefaction were significant features by using Logistic regression analysis. The corresponding regression equation In (p/(1 − p) = -3.676+2.919 internal echo +3.029 liquefaction +4.346 age).

**Conclusion:**

Internal echo, age, and liquefaction are independent ultrasound characteristics in predicting the malignancy of PTBs.

## Introduction

Phyllodes tumors of the breast (PTBs) are rare fibroepithelial tumors and account for less than 1% of all breast neoplasms [1, 2]. They are typically presented as a leaf-like pattern with combined features from both epithelial lesion and mesenchymal lesionin microscopy. Histologically, depending on their mitotic activity, stromal characteristics, and degree of cellular atypia, the biological behavioral gamut of PTBs ranges from benign, borderline to malignant [3]. The majority of PTBs are benign ones. Among the resected PTBs, benign, borderline and malignant PTBs accounted for 60%, 20%, and 20%, respectively [4, 5]. Surgery is the standard treatment of PTB. Recurrence rates of borderline and malignant PTBs are relatively higher than benign ones [6]. Wide excision of all types of phyllodes is necessary to avoid local recurrence and subsequent surgery, especially for borderline and malignant ones [7, 8]. Furthermore, malignant PTB can metastasize to the lungs, bones, and pleura, which may have fatal effects on patients [9]. Therefore, the pre-operation diagnosis is important for the treatment of PTB.

Ultrasonography (US), mammography, and magnetic resonance imaging (MRI) are considered as the main pre-operational diagnostic methods for PTB. According to previous studies, mammography was inconclusive for differentiating the pathologic grades of PTBs [10, 11]. Several MRI findings or parameters, such as low apparent diffusion coefficient (ADC) and tumor signal intensity, can be used to help determine the histologic grade of PTB [12–14]. However, there are very limited studies available focusing on the US characteristics of PTB [15, 16].

To our knowledge, no studies had been conducted to investigate the possible correlations between US findings and the histologic grades of PTB. In this study, we propose to investigate the capability of US characteristics in predicting benign (pathologically classified as benign PTB) and malignant PTB (pathologically classified as borderline or malignant PTB).

## Materials and methods

### Patients

This study was approved by the Institutional Review Board and Ethics Committee of Sichuan Cancer Hospital (SCCHEC-03-2017-010). All patients signed written informed consent before the examination.

From January 2017 to September 2020, a total of 79 consecutive patients with 79 lesions were enrolled in this study. The inclusion criteria were as follows: (1) all PTB should be classified as different risk levels by post-operative pathology (i.e. benign, borderline, or malignant); (2) patients’ medical history and US images were all complete and comprehensive. The exclusion criteria were as follows: (1) unsatisfied US image quality or incomplete vascular information; (2) recurrent PTB were not enrolled in this study.

### Equipments

Aixplorer US diagnostic imaging system (SuperSonic Imagine, Aixen Provence, France),Philips iU Elite, Epiq5 system (Philips Medical Systems, Bothell, WA), GE Logiq E9, and Mylab Twice were used. All systems were equipped with a high-frequency linear array probe (4–13 MHz) and a convex transducer (1–5 MHz).

### US characteristics of PTB

The US characteristics of PTB in this study include shape (regular or lobulated), margin (well defined or ill-defined), size (the largest diameter of the lesion), internal echo (homogeneous or heterogeneous), vascularity (poor: Adler classification 0-II; or abundant: Adler classification III) and liquefaction (present or absent).

### Statistical analysis

Categorical data were presented with frequency and percentage. Fisher’s exact test was used to compare categorical variables. Continuous data were presented with mean and standard deviation. Receiver operating characteristic (ROC) curves analysis was performed to estimate the capability of different variables in predicting the malignancy of PTB. A multivariate logistic regression model, including statistically significant risk factors in predicting the malignancy of PTB, was also established.

## Results

A total of 79 cases of PTB were reviewed in this study, including 39 benign and 40 malignant ones. Patients with malignant PTB were elder than those with benign PTB (48.0 vs. 34.8, p < 0.001). Compared with benign PTB, the lesion size of malignant PTB was larger (3.19cm vs. 5.3cm, p < 0.001). Moreover, malignant PTBs were presented in lobulated shape, with heterogeneous inner echo as well as abundant vascularity in the tumor. In addition, malignant PTBs showed more liquefactions in the tumor than benign ones (Table 1).

**Table 1:** Characteristics comparison between malignant and benign PTBs.

	Benign PTB	Malignant PTB	*P* value
Age	34.8±10.3	48.0±9.2	0.0001
Size (cm)	3.19±1.94	5.32±3.42	0.001
Margin well-defined ill-defined)			0.003
well defined	25	12	
ill-defined	14	28	
Shape			0.001
regular	24	10	
lobulated	15	30	
Internal-echoecheeechoechoheterogeneous)			0.0001
homogeneous	31	6	
heterogeneous	8	34	
Liquefaction			0.0001
absent	38	16	
present	1	24	
Vascularity			0.007
poor	33	22	
abundant	6	18	

**Fig 1:**
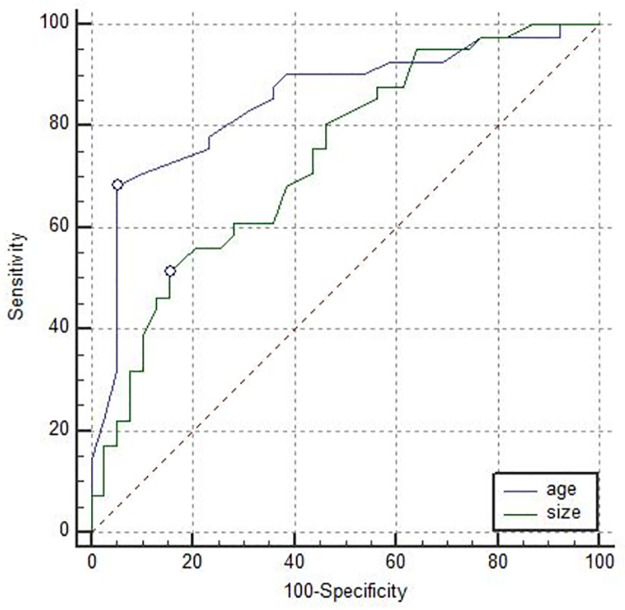
Receiver operating characteristic (ROC) curves for age (AUC:0.85; cut-off value: 46.5 years old), size (AUC:0.74; cut-off value:4.45cm).

In ROC curve analysis, the area under the curve (AUC) for patient age and lesion size were 0.85 and 0.73, separately. The optimal cut-off value was 4.45cm for lesion size and 46.5 for patient age (Fig 1) (Table 2). The age had a higher AUC in the differential diagnosis of PTBs compared with lesion size. However, the difference was not statistically significant (0.85 vs 0.73, *P* = 0.1).

**Table 2:** Diagnostic performance of age and size in differentiating PTBs.

	Cut-off value	AUC	Sen(%)(95%CI)	Spe(%)(95%CI)
age	46.5	0.85	68.3(51.9,81.9)	94.9(82.7,99.4)
size	4.45	0.74	51.2(35.1,67.1)	84.6(69.5,94.1)

**Fig 2:**
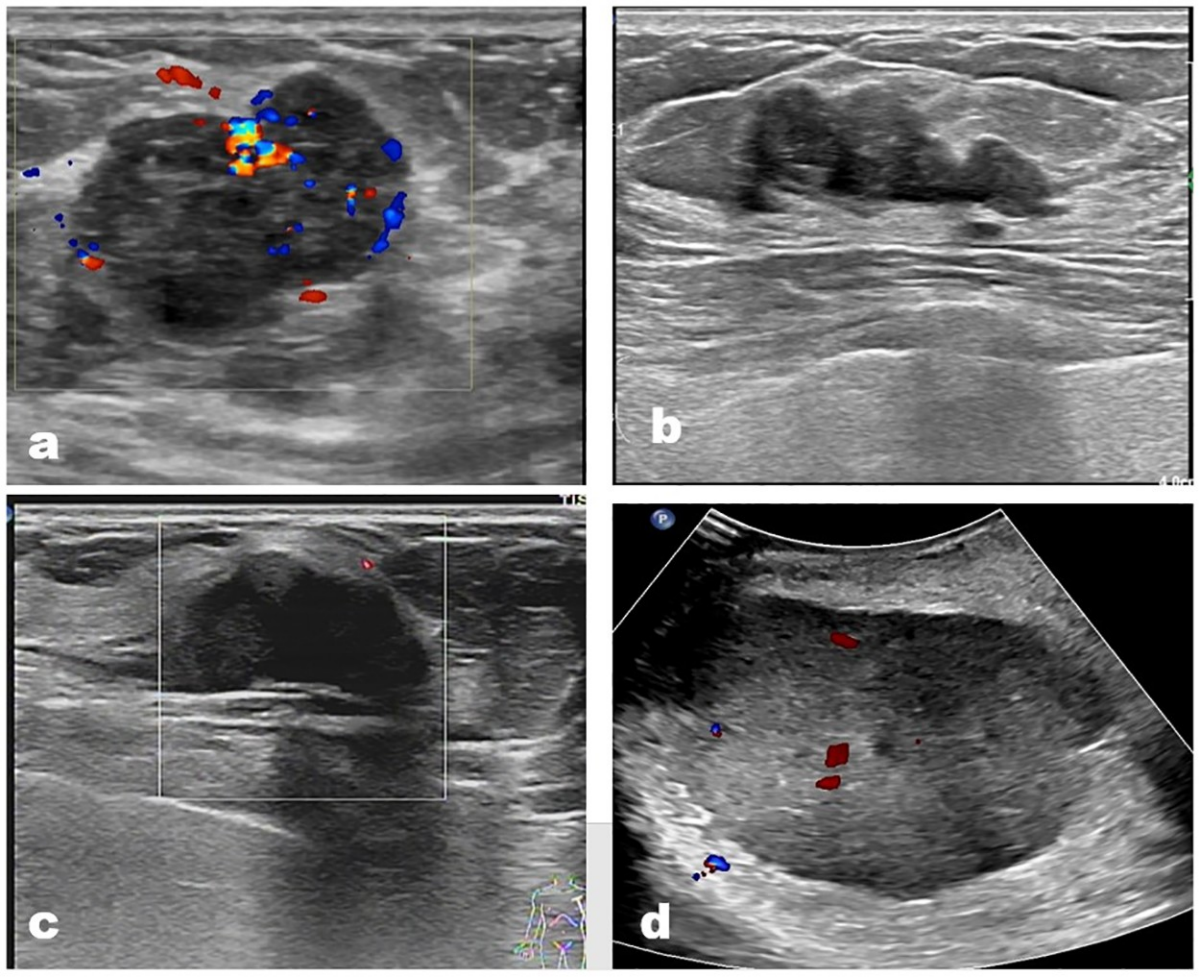
US images of benign PTBs. a) A 44 years old woman with a 3.2cm PTB in her left breast; b) A 32 years old woman with a 2.6cm PTB in her right breast; c) A 52 years old woman with a 2.6 cm PTB in her left breast; d) A 28 years old women with a 9.6cm PTB in her right breast. All these images showed regular tumors with homogeneous inner echo, without liquefaction in the tumor (even in a 9.6cm tumor).

**Fig 3:**
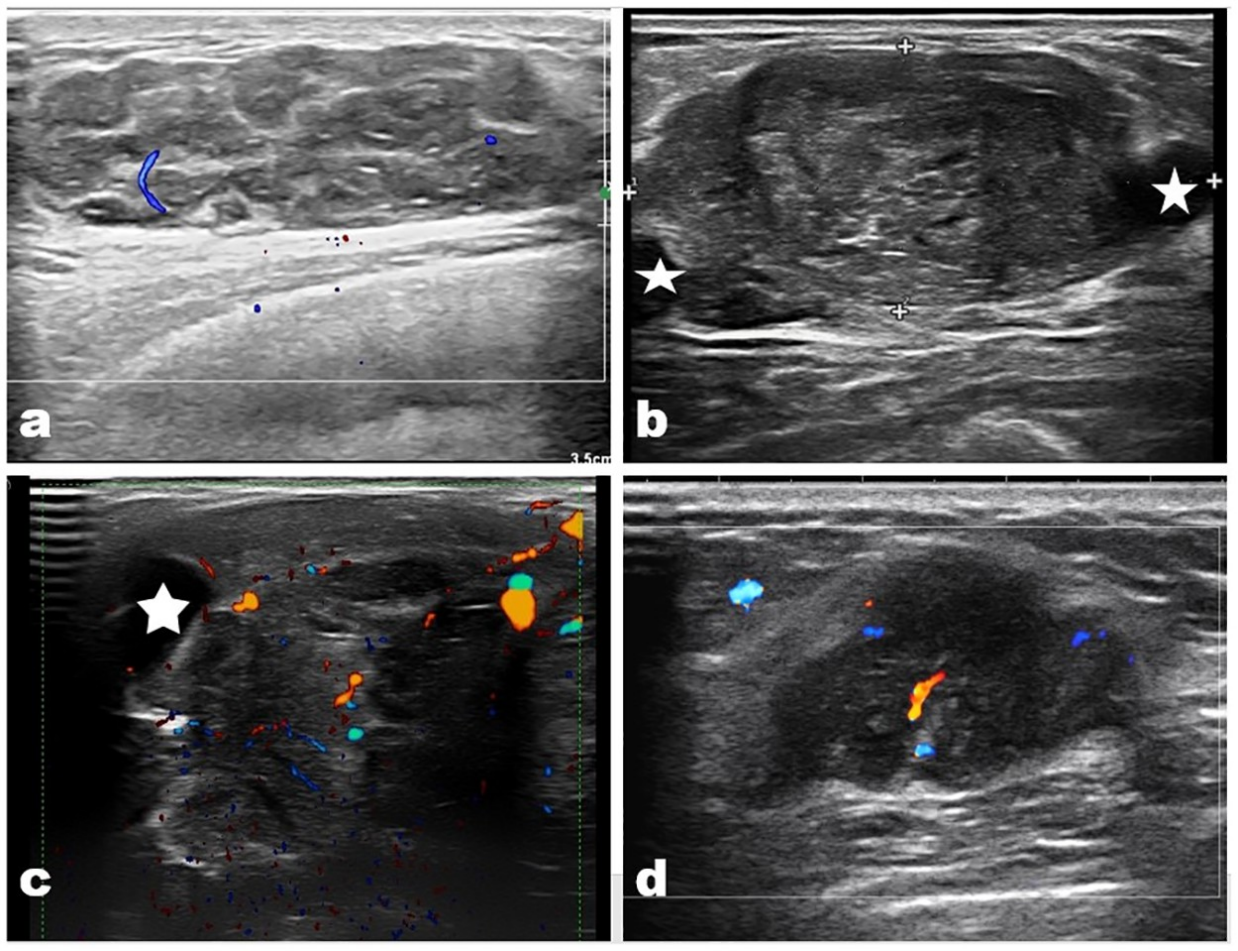
US images of malignant PTBs. a) A 47 years old woman with a 4.4cm PTB in her left breast; b) A 40 years old woman with a 4.7cm PTB in her left breast; c) A 58 years old woman with a 10.4 cm PTB in her right breast; d) A 60 years old women with a 3.3cm PTB in her right breast. All these images showed irregular tumors with heterogeneous inner echo, with liquefaction in the tumor (star). Patients’ age is elder than that of benign PTB patients.

In multivariate logistic regression, age (>46.5, 95% confidence interval [CI] 7.14–834.3), liquefaction (with liquefaction, 95% CI 1.92–222.5), and inner echo (heterogeneous, 95% CI 1.93–177.9) emerged as independent predictors for differentiating between malignant and benign PTB (Table 3) (Figs 2 and 3).

**Table 3:** Multivariate analysis with significant factors.

	Reference	Odds ratio	95%CI	*P* value
Internal echo	heterogeneous	2.919	(1.93,177.95)	0.01
Liquefaction	With liquefaction	3.029	(1.92,222.47)	0.01
Age	>46.5	4.346	(7.14,834.32)	0.00

## Discussion

Phyllodes tumor is a rare breast tumor with heterogeneous fibroepithelial. It constitutes a wide range of tumors, from benign to malignant [17]. A higher recurrence rate is observed in borderline and malignant tumors, which also had a higher tendency to systemic metastasis compared with benign ones. Due to various biological behaviors of PTB, wider surgical excision is needed especially for borderline and malignant PTB, which may further require chemotherapy and adjuvant radiation therapy treatment [18]. Given these reasons, a preoperative diagnosis is vital for the choice of PTB treatment. However, there are very limited related studies available investigating different imaging features between benign and malignant PTBs.

According to previous studies, benign PTB is more common and accounts for 60–75% of all PTBs [19, 20]. The percentage of malignant PTB in our study, however, was slightly more than that of benign one (51%vs.49%), which is inconsist with prior studies. This difference might relate to the fact that most cases in our study were categorized with high BI-RADs category before surgery (30 cases were categorized as BI-RADS 3, 49 cases were 4 and above). Nearly half of the patients enrolled in our study were referred from other hospitals or institutions for a second diagnostic opinion, meaning that they had been screened and had a high suspicion of malignancy.

Most patients affected by PTB were between the ages of 35 and 55. The overall mean age of patients in our study was 41.4 years, with malignant tumors developed later than benign ones (48.0 years vs. 34.8 years). There was a statistical difference in patient age between benign and malignant tumors (p < 0.001). The lesion size of these two types of tumors is also statistically different, where malignant PTB had a larger size (3.19cm vs. 5.3cm, p < 0.001) than benign one. These findings concur with previous literatures that malignant PTB is associated with larger lesion size and elder patient age [21]. US characteristics associated with tumor types, including shape, margin, internal echo, vascularity and liquefaction were also investigated. All these US characteristics turned out to be statistically different between malignant and benign tumors. To summarize, we found that malignant PTBs were presented in lobulated shape, with heterogeneous inner echo as well as abundant vascularity in the tumor. In addition, there were more liquefactions in malignant tumors than in benign ones.

Some previous studies have also noted that irregular masses with non-circumscribed margins might be related to malignant PTB. The complex cystic echogenicity was found to be helpful in the differentiation of malignant and benign phyllodes tumors [15, 22]. However, the differences in internal echo and vascularity between malignant and benign PTB have not been noted yet.

We proposed that, for lesion size, the optimal cut-off value between benign and malignant was 4.45cm, with a sensitivity of 51.2% and a specificity of 84.6%, respectively. Liberman et al. noted that phyllodes tumors larger than 3 cm had a higher likelihood of being malignant [10]. Megan et al. identified phyllodes tumor larger than 7 cm as an indication of an increased likelihood of being malignant [22]. Our threshold is in the middle of these previous results. In our series, half of the benign PTBs were less than 3cm in lesion size (21/39, 53.8%), and only 20% (8/40) of the malignant PTBs were with lesion size larger than 7cm. Therefore, the threshold we proposed would be more meaningful in malignancy diagnosis. Moreover, the cut-off value of patient age we proposed in this study was 46.5 years, with a sensitivity of 68.3% and a specificity of 94.9%, respectively. This cut-off value is younger than a previous study, which reported that patients older than 55 indicated a higher likelihood of malignant phyllodes tumors [22].

In our study, according to results of stepwise LR, age (>46.5), liquefaction (with liquefaction), and heterogeneous inner echo emerged as independent risk factors for malignant PTB. Previous studies had productive discussions on the correlation between patient age and the pathological grade of PTB, but no consistent conclusions have been drawn. Some studies suggested that the age of patients was not related to the pathological grade of PTB, but other relevant studies believed that malignant PTB was more common in elderly patients aged from 45 to 54 years old. The latter studies found that the increase of age was related to the increase of the pathological grade of PTB, which is similar to our findings. At present, few studies have reported on the correlations between internal echo and PTB malignancy. The findings of our study indicated that the number of cases of malignant PTB with inhomogeneous internal (34/40) was much higher than that of benign PTBs (8/39). In other words, heterogeneous inner echo helped distinguish benign from malignant phyllodes tumors. This finding is similar to the results of the study by Chao et al. [16].

Liquefication was also associated with malignant PTB in our findings. Yabuuchi’s study confirmed that the cystic change on MRI can help determine the histologic grade of breast PTB [12]. Other studies also reported that malignant PTB demonstrated with more cystic space and heterogenous inner echo on ultrasound [23, 24]. Our study showed that liquefication, which is probably caused by the continuous and rapid growth of tumor cells and stromal cells, can be considered as an independent risk factor for malignant PTB.

This study had some limitations as well. First, this is a retrospective study with a relatively small sample size. Current results showed that US characteristics such as size and shape had no significance in differentiating between malignant and benign PTBs. All these might be further evaluated with greater sample sizes and a longer follow-up period. Meanwhile, a large-scale prospective study is required to verify the clinical value of our prediction model.

## Conclusion

In conclusion, the internal echo, age, and liquefaction can be used for a noninvasive risk level prediction model for PTB. This model may help identify the different pathological grades of PTB before surgery.

## Supporting information

S1 Data (XLSX)
